# Reliability and validation of an electronic penicillin allergy risk-assessment tool in a pregnant population

**DOI:** 10.1186/s13223-024-00918-3

**Published:** 2024-10-19

**Authors:** Joanne Wang, Chelsea Elwood, Vanessa Paquette, Natasha Kwan, Stephanie Erdle, Melissa Watt, Julie Van Schalkwyk, Jeffrey N. Bone, Ashley Roberts, Raymond Mak, Tiffany Wong

**Affiliations:** 1 Division of Allergy and Immunology, Department of Medicine, University of British Columbia, Vancouver, BC Canada; 2 Department of Obstetrics and Gynecology, University of British Columbia, Vancouver, BC Canada; 3 Department of Pharmacy, Children’s and Women’s Health Centre of British Columbia, Vancouver, BC Canada; 4 Division of Allergy and Immunology, Department of Pediatrics, University of British Columbia, Vancouver, BC Canada; 5 Women’s Health Research Institute, BC Women’s Hospital, Vancouver, BC Canada; 6 BC Children’s Hospital Research Institute, University of British Columbia, Vancouver, Canada; 7 Division of Infectious Diseases, Department of Pediatrics, University of British Columbia, Vancouver, BC Canada

**Keywords:** Penicillin allergy, Penicillin de-labelling, FIRSTLINE, PENFAST, JAMA tool

## Abstract

**Background:**

Penicillin allergy adversely impacts patient care, yet most cases do not have true allergies. Clinicians require efficient, reliable clinical tools to identify low risk patients who can be safely de-labeled. Our center implemented the FIRSTLINE electronic point-of-care decision support tool to help non-allergist practitioners risk stratify patients with penicillin allergy. We sought to explore the reliability and validity of this tool in relation to allergist assessment and actual patient outcomes. We additionally compared it with two other published stratification tools, JAMA and PENFAST, to assess ability to accurately identify low risk patients appropriate for direct oral challenge.

**Methods:**

In this single-center, retrospective, observational study, 181 pregnant females with self-reported penicillin allergy between July 2019 to June 2021 at BC Women’s Hospital, Vancouver, Canada were used to assess the reliability and validity of all three tools. Physician-guided history of penicillin use and symptoms were used for scoring. Results and recommendations were compared to actual patient outcomes after clinician decision for direct oral challenge or intradermal tests. We compared the performance of JAMA, PENFAST and FIRSTLINE.

**Results:**

181 patients were assessed. 176/181 (97.2%) patients were deemed not allergic. Each risk stratification tool labelled majority of patients as low risk with 88.4% of patients PENFAST 0–2, 60.2% of patients JAMA low risk, 86.7% of patients FIRSTLINE very low risk.

**Conclusion:**

We demonstrate that our point-of-care electronic algorithm is reliable in identifying low risk pregnant patients, as compared to an allergist assessment. To our knowledge, this is the first study to provide direct comparison between multiple decision support tools using the same population, minimizing participant bias. Providing clinical algorithms to risk stratify patients, can enable healthcare professionals to safely identify individuals who may be candidates for direct penicillin oral challenges versus needing referral to specialists. This increases the generalizability and efficiency of penicillin allergy de-labeling.

**Supplementary Information:**

The online version contains supplementary material available at 10.1186/s13223-024-00918-3.

## Background

Unconfirmed beta-lactam antibiotic allergies adversely impact patient care as using alternative antibiotics leads to increased risk of multi-drug resistant bacterial infections, prolonged hospitalization, and greater healthcare utilization [1]. Reported international penicillin allergy prevalence is approximately 10%, with most cases not having clinically significant hypersensitivity [2]. A systematic review revealed 96.5% of patients could be de-labeled without serious adverse reactions [3]. This is further supported by recent evidence whereby low risk penicillin allergy patients can safely receive direct oral challenge [4]. Our group previously described safety of DOC in pregnant people with low risk penicillin allergy histories [5]. Investigating penicillin allergy can be time and resource intensive, including an allergist referral, intradermal skin testing (IDT), and subsequent direct oral challenge (DOC).

Due to the high prevalence of reported penicillin allergy and limited allergist availability, non-allergist clinicians require an efficient, reliable approach to identify low risk patients who can be safely de-labeled. We have previously published a study showing that various healthcare professionals were able to safely and accurately risk stratify penicillin allergies with an electronic clinical algorithm in pediatric patients [6]. The electronic algorithm has been adapted for clinical use through an electronic decision support tool hosted by an antimicrobial stewardship mobile platform: FIRSTLINE (https://app.firstline.org/en/clients/39-bc-womens-hospital/steps/6158). Other risk stratifying algorithms exist, including: JAMA toolkit, a manual stratification and management tool, and PENFAST, a four-question tool providing the risk of true penicillin allergy (Supplementary material) [6–8].

For the pregnant population, many are hesitant to proceed with penicillin allergy de-labeling despite previous published literature on the safety of DOC during pregnancy [9]. To our knowledge there has been no specificity nor sensitivity comparison between the various de-labeling tools to inform practitioners on their ability to effectively identify low risk individuals who would qualify for a direct oral challenge test.

In this study, we sought to explore the reliability and validity of our electronic point-of-care decision support tool compared to an allergist assessment of “low risk”. We additionally compared the ability of the decision support tool to two other published stratification tools to identify patients at low risk of true penicillin allergy: JAMA, and PENFAST, to assess their ability to accurately identify low risk patients appropriate for direct oral challenge. We hypothesized that our electronic tool would be reliable and have high specificity for identifying low risk patients when compared with other decision support tools.

## Methods

This is a single-center, retrospective, observational study with 181 pregnant females between July 2019 to June 2021. Patients with reported penicillin allergy were referred to the BC Women’s Hospital Penicillin Allergy clinic in Vancouver, Canada and were included regardless of medical, surgical, or medication history. Self-reported allergy histories required for each tool were routinely collected prior to patients’ first clinic visit. Information was stored on REDCap. This cohort was included in previous studies investigating the safety of DOC in pregnant populations [9].

The primary outcome of this study was to assess reliability of the electronic decision support tool compared with allergist assessment. Allergist assessment and to either pursue IDT and gold standard of DOC were previously recorded. Allergy histories were applied to the electronic decision support tool and the risk categories decided by the clinical assessment of the electronic decision tool were compared with allergist assessment. Risk categories included: allergic, very low risk of allergy, possible allergy, not allergic. The secondary outcome was to compare our electronic decision support tool with other published assessment tools to identify low risk patients appropriate for DOC. Patients were risk stratified by each tool into high, intermediate, and low risk categories.

With our interest specifically to identify patients at lowest risk for penicillin allergy, we opted to categorize all patients in the low risk stratification tier of each tool as screen negative this included those scoring PENFAST = 0–2 (very low and low risk), JAMA = low risk, FIRSTLINE = very low risk. All remaining patients were considered test screen positive. All patients deemed very low risk by clinician judgment received DOC. All remaining patients received IDT with benzylpenicilloyl polylysine and penicillin G. Positive IDT is defined as 3 × 3 mm greater than the negative control or a wheal size > 5 mm with underlying erythema, interpreted at 15 minutes [10–12]. If IDT was negative, patients then proceeded to a DOC. All equivocal IDT and delayed skin rash were considered test positive. All equivocal DOC without specifiers of exact symptoms experienced by patients were considered test negative. Examples of delayed DOC were those with delayed rash or delayed emesis (Type 4 hypersensitivity). None of the equivocal reactions had documented symptoms.

The assessor was blinded to patient information during data analysis. Collection of our data as a prospective cohort had received institution ethics approval. The screening tool results were then compared to clinician judgement to IDT and DOC. Additional ethics and consent waiver from the University of British Columbia was obtained for utilizing the data for algorithm validation.

## Results

The study included 181 pregnant females between 22 and 48 years-old, referred by obstetricians, general practitioners, registered midwives, or pharmacists (Supplementary material). Patient reported reaction histories were collected (Table 1). Most patients had reactions over ten years ago (80.1%) and could not recall which penicillin drug was used (30.3%), nor the details of their reactions. The most reported adverse reactions were maculopapular rashes (49.2%), followed by urticaria (32.6%). Reaction histories were applied to the electronic de-labeling tool in order to obtain a risk category. Based on the tool recommendations: 7 patients screened as very low risk from the tool proceeded with a IDT based on clinician judgment while the remaining 150 patients with a DOC, while all 11 patients labeled as possible risk proceeded with DOC. Overall, 20 patients received IDT prior to DOC (Table 2). Of this, one patient (0.6%) had a positive result from an equivocal IDT. 161 (89.0%%) patients who had low risk assessments or moderate risk assessments and subsequent negative skin testing received DOC. Of this, four patients had delayed rashes and were deemed allergic. All of these four patients experienced skin rashes with two documented as morbilliform rash, none with documented time from oral challenge to adverse reaction (Table 3). In total, five patients were deemed allergic, however, none experienced anaphylaxis. Four patients with mild symptoms thought to be unrelated to the DOC were considered not allergic. One patient with equivocal DOC with repeated delayed emesis was thought secondary to pregnancy was also considered not allergic. Overall, 176 (97.2%) patients were deemed not allergic by an allergist. Patients were scored on each de-labeling tool (Table 4).

**Table 1:** Patient phenotype based on self-reported penicillin history. *Patients presented with a combination of symptoms.+Patients include those with delayed OC and equivocal ST. ^a^Reactions already prior: no details documented by patients. ^b^Other symptoms including: face swelling, teeth discoloration, cold sensation, blurred vision, subjective weakness, feet swelling, canker sore, throat discomfort, lost voice, headache, HSP. ^c^Other treatments included: Epinephrine: 0, antihistamines: 22, salbutabmol: 0, IV fluids: 1, steroids: 2, other: 12 (given another steroid, topical steroids, calamine lotion, unclear)

			Number of total patients (%)	Patient test negative (%)	Patient test positive^+^ (%)
Total Number of patients			181	176	5
Median age, median, (IQR)			34.5	35	34
Referral source		General practitioner	23 (12.7%)	22 (12.5%)	1 (20%)
		OBGYN	52 (28.7%)	51 (30.0%)	1 (20%)
		Registered midwife	85 (47.0%)	83 (47.1%)	2 (40%)
		Other	12 (6.6)	11 (6.3%)	1 (20%)
		Blank	9 (5%)	9 (5.1%)	0 (0%)
Type of penicillin used		Amoxicillin	50 (27.6%)	48 (27.3%)	2 (40%)
		Amoxicillin-Clavulanate	2 (1.1%)	1 (0.5%)	1 (20%)
		Don’t know	56 (30.3%)	56 (31.8%)	2 (40%)
		Other penicillin	73 (40.3%)	73 (41.5%)	0 (0%)
Time of reaction		> 10 years ago	145 (80.1%)	142 (80.1%)	3 (60%)
		> 5 years ago	16 (8.8%)	16 (9.1%)	2 (40%)
		13mo to 5 years ag0	8 (4.4%)	8 (4.5%)	0 (0%)
		7 to 12 months ago	1 (0.5%)	1 (0.5%)	0 (0%)
		3 to 6 months ago	1 (0.5%)	1 (0.5%)	0 (0%)
		Don’t know	10 (5.5%)	10 (5.7%)	0 (0%)
Number of doses till reaction onset		> 7 days	4 (2.2%)	4 (2.3%)	0 (0%)
		4 to 7 days	10 (5.5%)	9 (5.1%)	1 (20%)
		1 to 3 days	29 (16.0%)	29 (16.5%)	0 (0%)
		1 dose	23 (23%)	22 (12.5%)	1 (20%)
		Don’t know	110 (60.8%)	107 (60.8%)	3 (60%)
		Reaction already present prior^a^	1 (0.5%)	1 (0.5%)	0 (0%)
		Blank (missing information)	4 (2.2%)	4 (2.3%)	0 (0%)
Time of symptom onset after most recent dose		< 1 h	14 (7.7%)	13 (7.4%)	1 (20%)
		1 to 2 h	4 (2.2%)	3 (1.7%)	1 (20%)
		3 to 12 h	12 (6.6%)	12 (6.8%)	0 (0%)
		13 to 24 h	11 (6.1%)	11 (6.3%)	0 (0%)
		> 24 h	1 (0.5%)	1 (0.5%)	0 (0%)
		Don’t know	137 (75.7%)	134 (77.8%)	3 (60%)
		Symptom already present prior^a^	1 (0.5%)	1 (0.5%)	0 (0%)
		Blank (missing information)	1 (0.5%)	1 (0.5%)	0 (0%)
Patient reported reaction*	Skin	Macular/papular rash	89 (49.2%)	88 (50%)	1 (20%)
		Urticaria	59 (32.6%)	56 (31.8%)	3 (60%)
		Angioedema	11 (6.1%)	11 (6.3%)	0 (0%)
		Blistering/peeling skin or mucous membrane	1 (0.5%)	1 (0.5%)	0 (0%)
		Generalized pustulosis	0 (0%)	0 (0%)	0 (0%)
		Erythema multiforme	0 (0%)	0 (0%)	0 (0%)
	Respiratory	Cough	0 (0%)	0 (0%)	0 (0%)
		Wheeze	3 (1.7%)	3 (1.7%)	0 (0%)
		Stridor	0 (0%)	0 (0%)	0 (0%)
		Breathing difficulties	6 (3.3%)	6 (3.4%)	1 (20%)
	Gastrointestinal	Nausea	3 (1.7%)	3 (1.7%)	0 (0%)
		Vomiting x1	8 (4.4%)	8 (4.5%)	0 (0%)
		Vomiting multiple times	2 (1.1%)	2 (1.1%)	0 (0%)
		Abdominal discomfort	0 (0%)	0 (0%)	0 (0%)
		Diarrhea	1 (0.5%)	1 (0.5%)	0 (0%)
	Other	Palpitations	0 (0%)	0 (0%)	0 (0%)
		Decreased level of consciousness	1 (0.5%)	1 (0.5%)	0 (0%)
		Arthritis/arthralgia	2 (1.1%)	2 (1.1%)	0 (0%)
		Unexplained fever	1 (0.5%)	1 (0.5%)	0 (0%)
		Liver or kidney involvement	0 (0%)	0 (0%)	0 (0%)
		Other^b^	20 (11.0%)	20 (11.4%)	0 (0%)
Treatment given		No	65 (35.9%)	64 (36.4%)	1 (20%)
		Yes^c^	31 (17.1%)	29 (16.5%)	2 (40%)
		Don’t know	83 (45.9%)	81 (46.0%)	2 (40%)
		Blank (missing information)	2 (1.1%)	2 (1.1%)	0 (0%)

**Table 2:** Penicillin allergy test results. Result + are those who are deemed penicillin allergic, result- are those who are deemed penicillin nonallergic

	Result +	Result -	Total
Intradermal test	1 (0.6%)	19 (10.5%)	20
Oral challenge	4 (2.2%)	157 (86.7%)	161
Total	5	176	181

**Table 3:** Demographic information on patients who experienced delayed oral challenge reaction

	Patient 1	Patient 2	Patient 3	Patient 4
Type of delayed response documented	Delayed rash	Generalized morbilliform eruption	Delayed rash	Delayed morbilliform rash
FIRSTLINE score	Very Low risk (R2NC)	Very Low risk (R2NC)	Very Low risk (R2NC)	Very Low risk (R2NC)
PENFAST Score	1	0	1	1
JAMA Score	Low	Medium	Medium	Low
Previous Penicillin allergy history	> 10 years	> 5 years	> 5 years	> 5 years
Previous penicillin indication	Other	Urinary tract infection	Pneumonia	Upper respiratory tract infection
How many doses till reaction occurred	Don’t know	Don’t know	4–7 days	Don’t know
How soon after did reaction occur	Don’t know	Don’t know	3–12 h	Don’t know
Previous reaction Reaction	Don’t know	Urticaria	Urticaria	Macularpapular rash
Treatment provided for previous reaction	Don’t know	No treatment	Antihistamine	Don’t know

**Table 4:** Clinician decision to pursue intradermal skin test versus oral challenge compared to risk stratification via penicillin de-labeling tool

		Intradermal Skin Test (*n*, %)	Oral challenge (*n*, %)	Total (*n*, %)
PENFAST	≥ 3	6 (3.3%)	15 (8.3%)	21 (11.6%)
	0–2	14 (7.7%)	146 (80.7%)	160 (88.4%)
JAMA	High – medium	17 (9.4%)	55 (30.4%)	72 (39.8%)
	Low	3 (1.7%)	106 (58.6%)	109 (60.2%)
FIRSTLINE	High – possible	13 (7.2%)	11 (6.1%)	24 (13.3%)
	Very low	7 (3.9%)	150 (82.9%)	157 (86.7%)

Based on the above results, our electronic assessment tool was able to label 86.7% of patients as very low risk, compared to 60.2% for JAMA low risk, and 88.4% for PENFAST score 0–2. Of these stratified low risk patients 80.7%, 58.6%, and 82.9% proceeded with a DOC based on clinician judgement for PENFAST, JANA, and FIRSTLINE respectfully.

## Discussion

Congruent with previous data, we found low rates of true penicillin allergy in the pregnant population [13]. We acknowledge that our cohort has a low test positivity rate, as the purpose of our penicillin clinic is for “de-labeling”, thus is likely a referral bias towards lower risk patients. Many patients who were stratified as relatively higher risk by the allergist required skin testing but were still mostly deemed not allergic. The low prevalence of allergy in our cohort limits the ability for us to assess comparative efficacy at “ruling in an allergy”. It is difficult to assess the risk stratification capacity in this study as it includes mainly low-risk patients. Future validation should occur in larger cohorts across more varied risk levels.

Despite these limitations, clinical decision tools were designed to identify patients at low risk to facilitate safe de-labeling efforts. All three tools are aimed at helping the clinician conduct a penicillin de-labeling assessment. Our electronic tool provides specific question phrases which clinicians can ask while PENFAST and JAMA tools require clinicians to word their own questions. Our study shows that our electronic assessment tool is a reliable electronic tool to safely identify low risk patients similar to a board certified allergist assessment. The detailed and extensive algorithm likely contributes to its increased rates to identify low-risk patients, however, the time required may be more difficult to integrate into a busy practice. The high percentages of low-risk patients which later proceeded with oral challenge based on our electronic tool confirms our initial hypothesis and supports its use to DOC patients. We acknowledge that electronic tools do not necessarily replace physician judgment, as indicated by a proportion of patients stratified as low risk by the tool, who went on to receive IDT. There are multiple factors that can influence this decision, including economic and patient -related factors, such as anxiety.

Another limitation of this study is there may be recall bias while patients recollected past information for the questionnaire. The decision to label all equivocal IDT results was made to maximize safety in pregnant women. However, if a gold standard DOC had been completed, the patient may have tolerated it and been de-labeled. Additionally, we completed this validation study at a single centre with non-random recruitment, therefore extrapolating data to other populations requires caution.

To our knowledge, this is the first study to provide comparison between various available clinical decision support tools for penicillin allergy risk assessment. Our electronic tool has the benefit of both being freely accessible online and providing direct management recommendations. Use of this tool can empower non-allergist healthcare providers to safely identify and manage patients at low risk of true penicillin allergy. This allows for more patients to be de-labeled in community and hospital-settings more quickly and reduces waiting times for allergists to assess and manage patients who are at higher risk of true allergy.

## Conclusion

Highly specific de-labeling tools can identify low risk patients while preserving safety. This tool can empower non-allergist healthcare providers to manage low risk patients in a resource limited healthcare system. We herein provide an assessment of reliability and show validity for an electronic point-of-care decision support tool for patients with penicillin allergy. We additionally provide a comparative analysis between our electronic tool and two other published risk-stratification tools in pregnant populations.

## Electronic supplementary material

Below is the link to the electronic supplementary material.

Supplementary Material 1

## Data Availability

All clinical decision tools are readily available to public. The datasets used and/or analysed during the current study are available from the corresponding author on reasonable request.

## Abbreviations

IDT: Intradermal skin testing
DOC: Direct oral challenge
BCWH: BC Women’s Hospital
PPV: Positive predictive value
NPV: Negative predictive value
