# Supplementary material for: Reliability and validation of an electronic penicillin allergy risk-assessment tool in a pregnant population
Source: Allergy Asthma Clin Immunol. 2024 Oct 19;20:55. doi: 10.1186/s13223-024-00918-3 (PMC11490178; doi:10.1186/s13223-024-00918-3)
Supplement: Supplementary file 1 — Supplementary Material 1 [file 13223_2024_918_MOESM1_ESM.docx]

**Supplementary Material**

| Variable | |  |
| --- | --- | --- |
| Median age, median, (IQR) | | 34.5 (5) |
| Gestational age, median (IQR) | | 39.0 (3) |
| First pregnancy, n (%) | | 94 (51.9%) |
| Referral source, n (%) | | GP: 23(12.7%), OBGYN 52(28.7%), other 12(6.6%), Registered midwife 85(47.0%), blank 9(5.0%) |
| Comorbidities, n (%) | Any comorbidities | 104 (57.5%) |
|  | Hypothyroid | 10 (5.5%) |
|  | Anxiety/Depression | 38 (21.0%) |
|  | Hypertension | 4 (2.2%) |
|  | Preeclampsia | 3 (1.7%) |
|  | Gestational diabetes, n (%) | 19 (10.5%) |
|  | Diabetes Mellitus | 0 (0%) |
|  | Migraines | 23 (12.7%) |
|  | Other | 42 (23.2%) |
| Current smoker, n (%) | | 0 (0%) |
| Current alcohol, n (%) | | 1 (0.6%) |

Supplementary Material Table 1.  Patient demographic. Value for age, gestational age are listed by median with interquartile range (IQR). Referral, gestational diabetes, current smoking, current alcohol use are listed by number of patients (percentage)

| Penicillin De-labeling Tool | Description |
| --- | --- |
| PENFAST | Four question clinical decision tool which risk stratifies patients to inform clinicians on rate of positive penicillin allergy test based on its five-point scoring system. |
| FIRSTLINE | Mobile platform for penicillin risk assessment with point of care answer entry based on patient history, providing immediate risk assessment and management recommendations to de-label low risk patients without need for diagnostic testing or allergist assessment. |
| JAMA | Risk stratification table based on patient history and symptoms, providing immediate risk assessment and management recommendations including direct oral challenge, skin prick test, and referral to allergist. |

Supplementary Material Table 2. Summary description of each penicillin de-labeling tool.

**PEN-FAST**^1^

Questions

1.PEN - Penicillin allergy reported by patient

2.F - Five years or less since reaction

3.A - Anaphylaxis or angioedema

4.S - Severe cutaneous adverse reaction

5.T - Treatment required for reaction

**FIRSTLINE**^2^


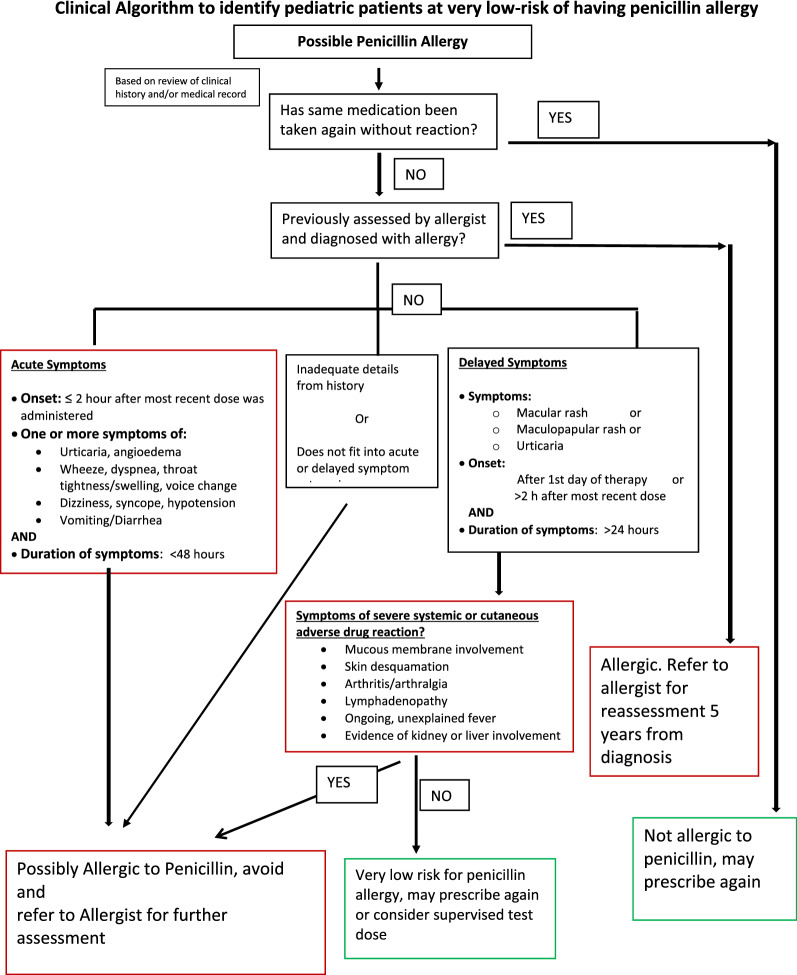


**Adapted JAMA Table 3. Risk Stratification for Penicillin Allergy Evaluation**^3^

|  | Low Risk | Medium Risk | High Risk |
| --- | --- | --- | --- |
| History | Isolated reactions that are unlikely allergic (eg, gastrointestinal symptoms, headaches)  Pruritus without rash Remote (>10 y) unknown reactions without features of IgE^b^  Family history of penicillin allergy | Urticaria or other pruritic rashes Reactions with features of IgE but not anaphylaxis^b^ | Anaphylactic symptoms^c^  Positive skin testing Recurrent reactions Reactions to multiple β-lactam antibiotics |
| Action | Prescribe amoxicillin course or perform a direct amoxicillin challenge under observation^d^ | Skin test followed by amoxicillin challenge under observation if the skin test is negative^e.^ Consider allergy/immunology referral. | Allergy/immunology referral or desensitization |

^a^No penicillin allergy testing should be performed on patients with possible penicillin-associated severe cutaneous adverse reaction, hemolytic anemia, organ-specific reaction, drug fever, or serum sickness. Patients with unstable or compromised hemodynamic or respiratory status and pregnant patients should never be considered low risk.

^b^IgE features classically include cutaneous symptoms, such as itching, flushing, urticaria, and angioedema, but also involve respiratory system (rhinitis, wheezing, shortness of breath, bronchospasm), cardiovascular system (arrhythmia, syncope, chest tightness), and gastrointestinal system (abdominal pain, nausea, vomiting, diarrhea) symptoms.

^c^The most severe IgE-mediated reaction is anaphylaxis (eFigure 1 in Supplement 1). Allergy/immunology consultation is advised.

^d^Considering patient comfort level with trying penicillin again and whether resources exist for observation.

^c^If skin testing is not possible, a graded amoxicillin challenge can be considered for medium-risk histories. A graded challenge often requires administration of a one-tenth to one-fourth full dose of the desired drug and a 30- to 60-minute period of monitoring followed by administration of a full dose of the desired drug and a final 30- to 60-minute period of monitoring

1. Trubiano JA, Vogrin S, Chua KYL, et al. Development and Validation of a Penicillin Allergy Clinical Decision Rule. *JAMA Intern Med*. 2020;180(5). doi:10.1001/jamainternmed.2020.0403

2. Roberts H, Soller L, Ng K, et al. First pediatric electronic algorithm to stratify risk of penicillin allergy. *Allergy, Asthma and Clinical Immunology*. 2020;16(1):1-5. doi:10.1186/S13223-020-00501-6

3. Shenoy ES, Macy E, Rowe T, Blumenthal KG. Evaluation and Management of Penicillin Allergy: A Review. *JAMA - Journal of the American Medical Association*. 2019;321(2). doi:10.1001/jama.2018.19283
